# Molecular Detection of Multiple Antimicrobial Resistance Genes in *Helicobacter pylori*-Positive Gastric Samples from Patients Undergoing Upper Gastrointestinal Endoscopy with Gastric Biopsy in Algarve, Portugal

**DOI:** 10.3390/antibiotics14080780

**Published:** 2025-08-01

**Authors:** Francisco Cortez Nunes, Catarina Aguieiras, Mauro Calhindro, Ricardo Louro, Bruno Peixe, Patrícia Queirós, Pedro Castelo-Branco, Teresa Letra Mateus

**Affiliations:** 1 Faculty of Medicine and Biomedical Sciences, University of Algarve, 8005-139 Faro, Portugal; poqueiros@ulsalg.min-saude.pt (P.Q.); pjbranco@ualg.pt (P.C.-B.); 2 Serviço de Gastroenterologia, Unidade Local de Saúde do Algarve–Hospital de Faro, 8005-139 Faro, Portugal; catarina.maria@edu.ulisboa.pt (C.A.); bpeixe@chalgarve.min-saude.pt (B.P.); 3 Serviço de Medicina Interna, Unidade Local de Saúde do Algarve–Hospital de Portimão, 8500-338 Portimão, Portugal; mauro.santos@chalgarve.min-saude.pt; 4 Hospital Particular do Algarve–Unidade de Alvor, 8000-386 Faro, Portugal; louro_scout@hotmail.com; 5 Serviço de Gastroenterologia, Unidade Local de Saúde do Algarve–Hospital de Portimão, 8500-338 Portimão, Portugal; 6 Algarve Biomedical Center Research Institute (ABC Ri), University of Algarve, 8005-139 Faro, Portugal; 7 CISAS—Center for Research and Development in Agrifood Systems and Sustainability, Escola Superior Agrária, Instituto Politécnico de Viana do Castelo, 4900-437 Viana do Castelo, Portugal; 8 EpiUnit ITR, Instituto de Saúde Pública, Universidade do Porto, 4050-091 Porto, Portugal; 9 Veterinary and Animal Research Centre (CECAV), Associate Laboratory for Animal and Veterinary Sciences (AL4AnimalS), Universidade de Trás-os-Montes e Alto Douro, 5000-801 Vila Real, Portugal

**Keywords:** metronidazole, tetracycline, PCR, sequencing, AMR

## Abstract

**Background/Objectives**: *Helicobacter pylori* (*H. pylori*) is a common gastric pathogen linked to gastritis, gastroduodenal ulcers, and gastric cancer. Rising antimicrobial resistance (AMR) poses challenges for effective treatment and has prompted the WHO to classify *H. pylori* as a high-priority pathogen. This study aimed to detect the prevalence of AMR genes in *H. pylori*-positive gastric samples from patients in Algarve, Portugal, where regional data is scarce. **Methods**: Eighteen *H. pylori*-positive gastric biopsy samples from patients undergoing upper gastrointestinal endoscopy were analyzed. PCR and sequencing were used to identify genes associated with resistance to amoxicillin (*Pbp1A*), metronidazole (*rdxA*, *frxA*), tetracycline (*16S rRNA* mutation) and clarithromycin (*23S rRNA*). Sequence identity and homologies were verified using tBLASTx and the Comprehensive Antibiotic Resistance Database (CARD). **Results:** Out of the 18 *H. pylori*-positive samples, 16 (88.9%) contained at least one AMR gene. The most frequent genes were *rdxA* (83.3%) and *frxA* (66.7%) for metronidazole resistance, and the *16S rRNA* mutation (66.7%) for tetracycline. Resistance to amoxicillin and clarithromycin was detected in 27.8% and 16.7% of cases, respectively. Most samples (72.2%) had multiple resistance genes. A significantly strong association was found between female sex and the presence of the *rdxA* gene (*p* = 0.043). **Conclusions**: The study reveals a high prevalence of *H. pylori* resistance genes in Algarve, particularly against metronidazole and tetracycline. These findings highlight the need for local surveillance and tailored treatment strategies. Further research with larger populations is warranted to assess regional resistance patterns and improve eradication efforts.

## 1. Introduction

*Helicobacter pylori* (*H. pylori*) is a spiral-shaped, Gram-negative *bacterium* that colonizes the human gastric lining, and it is a major cause of chronic gastritis, peptic ulcer, and gastric cancer, such as gastric adenocarcinoma and mucosa-associated lymphoid tissue lymphoma (MALT) [1,2].

Globally, more than 50% of the population is estimated to be infected with *H. pylori*, although prevalence varies widely by region, socioeconomic status, and age [2]. In some developing countries, infection rates may exceed 80%, often acquired during childhood and persisting lifelong without treatment. In Europe, the prevalence has been reported to range between 30% and 50%, while in Portugal, recent data suggests rates between 48% and 70% depending on the region and diagnostic methods used [3,4,5,6,7]. The bacterium has developed sophisticated mechanisms to persist in the hostile acidic environment of the stomach, including the production of urease, which neutralizes gastric acid, and the expression of adhesins that facilitate attachment to gastric epithelial cells [1,2].

The emergence and spread of antimicrobial resistance in *H. pylori* is a multifactorial problem, strongly associated with inappropriate or repeated use of antibiotics, such as clarithromycin and metronidazole, both in eradication regimens and for other infections [8,9]. A 2018 European surveillance study demonstrated that clarithromycin resistance surpassed 15% in most participating countries, which is the threshold above which standard triple therapy is no longer recommended [3]. Moreover, the European Registry on *H. pylori* Management (Hp-EuReg) highlighted increasing resistance trends from 2013 to 2020, especially in Southern and Eastern Europe, raising concerns about treatment efficacy and the need for regional susceptibility data to guide empirical therapy [8].

AMR in *H. pylori* is particularly concerning because it directly undermines the efficacy of first-line and rescue therapies, leading to treatment failures, persistent infection, and increased risk of gastric malignancy [9,10]. The increasing resistance to clarithromycin, metronidazole, and levofloxacin, commonly used in eradication regimens, has contributed to a decline in overall eradication rates, especially in Southern European countries like Portugal [9,11,12,13,14]. Moreover, high AMR prevalence contributes to the need for longer treatment durations, more complex therapeutic combinations, and increased healthcare costs [2,15,16]. Therefore, rapid molecular diagnostics that target these key mutations are essential to tailor effective treatment regimens, reduce empirical therapy failures, and prevent further spread of resistant strains [15,16,17].

Despite its global prevalence, treatment of *H. pylori* infection has become increasingly challenging worldwide, due to rising antimicrobial resistance (AMR) [8,9]. As a result, the World Health Organization (WHO) has classified *H. pylori* as a high-priority pathogen for which there is an urgent need for new treatments, specifically concerning clarithromycin resistance an essential component of traditional eradication regimens [10,18,19]. This prioritization underscores the global concern regarding resistance trends and the necessity for ongoing surveillance and the development of novel antimicrobial strategies [10,18,19].

Standard *H. pylori* eradication treatment includes a combination of different antibiotics, such as clarithromycin, amoxicillin or metronidazole along with a proton pump inhibitor. However, the effectiveness of these treatments has significantly declined over the years, primarily due to the development of resistance to the commonly used antibiotics [16,20,21,22]. Current treatment guidelines emphasize a shift toward personalized or region-specific therapies based on local antibiotic resistance patterns and prior treatment history. Molecular testing for resistance, particularly to clarithromycin, is increasingly recommended to guide therapy selection [2,16]. Bismuth-containing quadruple therapy is often preferred in areas with high resistance rates or previous treatment failures [2,15,20]. Guidelines also highlight the importance of post-treatment eradication testing, typically using urea breath or stool antigen tests, to confirm successful therapy [8]. The growing role of primary care in managing *H. pylori* infection underscores the need for accessible diagnostics and standardized treatment protocols [2,15,16,20].

Meta-analyses show a worrisome global trend: clarithromycin resistance ranges from 17% to 30% across different WHO regions, while metronidazole resistance can exceed 50% in some areas, undermining the success of conventional treatment regimens [10]. The growing challenge of treatment failure emphasizes the importance of updating treatment guidelines, improving diagnostic methods, and implementing antibiotic stewardship programs to limit further resistance development [1,16].

In real-world clinical practice, as observed in several observational studies, many cases of *H. pylori* infection remain undiagnosed or are treated empirically without confirmation or follow-up testing [15,16,23]. The involvement of primary care services in the diagnostic and therapeutic process is increasing, reflecting the need for integrated care models that incorporate resistance surveillance, patient education, and evidence-based decision-making [16].

AMR can be associated with different mechanisms, and *H. pylori* has developed a diverse array of AMR mechanisms, making eradication increasingly difficult, it shows acquired resistance to several drugs such as tetracyclines, beta-lactams, nitroimidazoles, fluoroquinolones, macrolides, and rifampicin [9,17,20].

These resistance mechanisms are frequently driven by specific point mutations in bacterial genes. For example, mutations in the *23S rRNA* gene (such as A2142G, A2143G) are primarily responsible for clarithromycin resistance, while alterations in *rdxA* and *frxA* genes underlie metronidazole resistance. Similarly, mutations in the 16S rRNA gene can lead to tetracycline resistance, and changes in the pbp1A gene contribute to amoxicillin resistance [9,17,20,24].

*H. pylori* resistance to tetracyclines is mainly associated with mutations in the *16S rRNA* gene, particularly triple-base mutations in positions 926–928, disrupting tetracycline’s ability to bind the bacterial ribosome [24,25]. The main factor contributing to amoxicillin resistance in *H. pylori* is mutations in the *Pbp1A* gene, encoding penicillin-binding protein 1A and reducing binding affinity. *H. pylori* metronidazole resistance is due to mutations or inactivation involving the genes *rdxA* and *frxA* which encode nitroreductases essential for activating the prodrug form of metronidazole and [24,25] also from the overexpression of efflux pumps such as *hefA*, which belong to the resistance-nodulation-cell-division (RND) family [26]. Lee et al. (2018) demonstrated that metronidazole-resistant strains with intact *rdxA/frxA* genes still exhibited treatment failure due to efflux-mediated extrusion of the drug [26]. This implies that phenotypic testing alone may underestimate resistance levels, highlighting the importance of comprehensive genotypic profiling. Resistance to clarithromycin is due to point mutations in the peptidyl transferase region encoded in domain V of *23S rRNA*, and three mutations in this domain, A2141G/C and A2143G, respectively, are responsible for more than 90% of clarithromycin-resistant *H. pylori* strains [24,25]. Another mechanism involved is associated with multidrug efflux pump transporters, mainly the resistance-nodulation-cell-division [24,25]. These molecular mechanisms often acting synergistically, underline the need for rapid molecular diagnostics and resistance-guided therapy to manage *H. pylori* infections effectively [24,25].

Overall, metronidazole resistance can range from 23% to 56% across different WHO regions, while clarithromycin resistance can be over 30% in some regions. Regarding amoxicillin, its resistance is typically below 5% [10].

Several studies have shown that *H. pylori* AMR rates are of concern in Portugal, particularly regarding clarithromycin, metronidazole, and levofloxacin [12,13,14]. These studies evidence overall resistance rates of 42% for clarithromycin, 25% for metronidazole, 9% for ciprofloxacin, 10% for multidrug resistance to clarithromycin and metronidazole, and reported rare resistance to tetracyclines [12,13,14].

In addition to regional prevalence, global resistance trends have shown alarming increases, especially in pediatric populations and low- and middle-income countries, where surveillance and molecular diagnostic capacities remain limited. A global systematic review by Salahi-Niri et al. (2024) reported that among children, clarithromycin resistance exceeded 20% in more than half of the WHO regions, with metronidazole resistance reaching over 40% in some African and Southeast Asian countries [27]. Furthermore, gender and age have been recognized as potential influencers of AMR prevalence and treatment outcome. Women, especially in older age groups, have been found to experience higher rates of drug-resistant infections, which may also affect *H. pylori* treatment success [5,28,29]

Of interest, zoonotic implications have also been explored, suggesting the possible environmental reservoir of *H. pylori*-like organisms. Cortez Nunes et al. (2023) reported molecular detection of tetracycline and metronidazole resistance genes in *H. pylori*-like organisms found in pigs, raising questions about agricultural AMR gene transmission and One Health considerations [30]. While these findings have yet to be directly correlated with human infections, they illustrate the potential for cross-species transmission of resistance genes.

In summary, the rising burden of antimicrobial resistance in *H. pylori* necessitates a shift toward region-specific treatment regimens informed by molecular surveillance. This is particularly relevant in areas like Portugal, where clarithromycin resistance is notably high. Adoption of molecular methods and integration of global AMR databases into clinical workflows will be essential for tailoring treatment strategies, improving eradication rates, and mitigating the spread of resistance.

Molecular testing for resistance mutations, such as PCR-based assays, can guide personalized therapy by identifying the most effective antibiotics before treatment. Therefore, this study aimed to identify and determine the presence of antimicrobial resistance genes (ARG) associated with *H. pylori* infection in Algarve, Portugal.

## 2. Results

### 2.1. Detection of Genes Conferring Resistance to Antimicrobials

*H. pylori* PCR-positive samples from patients that underwent UGE in Algarve were tested for AMRs. Only PCR amplicons that showed sequence homology to known resistance genes through alignment analysis were considered true PCR-positive results (Figures S1–S5).

Out of the 18 *H. pylori* DNA PCR-positive samples tested, 16 (88.9%) were PCR-positive for genes conferring resistance to antimicrobials, 15 (83.3%) and 12 (66.7%) were found PCR-positive for the *rdxA* and *frxA* genes that can be associated with metronidazole resistance, respectively; 12 (66.7%) were found PCR-positive for the *16S rRNA* mutation gene conferring tetracycline resistance, and five (27.8%) were found PCR-positive for the *Pbp1A* gene associated with resistance to amoxicillin. Three were found PCR-positive for the *23S rRNA* mutation gene conferring resistance to clarithromycin, shown in Table 1. Overall, the presence of genes conferring resistance to antimicrobials was more prevalent in female patients, and a statistically significant strong association was found between being female and having the metronidazole *rdxA* gene (*p* = 0.043).

Of the 16 *H. pylori* DNA-positive samples that were also positive for ARGs, only three patients (16.6%) were *H. pylori* DNA-positive with a mono-resistance gene identified, whereas 13 had multiple resistance genes identified (Table 2).

### 2.2. Sequencing and Sequence Analysis of Positive PCR Products

ARGs were identified by aligning nucleotide sequences using tBLASTx against protein reference sequences from the CARD version 3.2.4 [31].

The PCR-positive samples for the *rdxA* gene showed an identity of the matching region of 92–100% with *H. pylori rdxA gene* mutation conferring resistance to metronidazole (ARO:3007055), the PCR-positive samples of the *frxA* gene showed an identity of the matching region of 81–100% with *H. pylori frxA* gene conferring resistance to metronidazole (ARO:3007059). The obtained sequences were also analyzed using the Resistance Gene Identifier (RGI) tool to predict resistomes based on homology and SNP models. The RGI analysis confirmed the presence of *H. pylori rdxA* and *frxA* mutations associated with resistance to metronidazole, showing different SNPs (R90K, A118T, C49T, T31E, Y62D, V7I) with an identity of the matching region ranging from 89.5–100% (ARO:3007059) (Figures S6–S9). The PCR-positive samples of the *16S rRNA* mutation gene showed an identity of the matching region of 93–100% with *H. pylori 16S rRNA* mutation gene conferring tetracycline resistance (ARO:3003510) (Figure S10), the PCR-positive samples of the *Pbp1A* gene showed an identity of the matching region of 94–98% with the *H. pylori Pbp1A* gene associated with resistance to amoxicillin (ARO:3007060) (Figure S11), and the PCR-positive samples of *23S rRNA* mutation gene showed an identity of the matching region of 80–99% with the *H. pylori 23S rRNA* mutation gene conferring resistance to clarithromycin (ARO:3004134).

## 3. Discussion

The studied ARGs were the ones associated with the most commonly used antimicrobials, according to the recommendations and guidelines [2,16,20,22]. So, all the PCR-positive to *H. pylori* DNA samples (18) were tested for ARGs, only samples yielding PCR amplicons that aligned with known resistance genes based on sequence homology (BLAST or CARD results) were classified as PCR-positive. The results showed a prevalence of ARGs of 88.9% (16/18), 7 were male and 11 were females, with 71.4% (5/7) of male and 100% (11/11) of female patients with PCR-positive results for ARGs. Furthermore, the alignment of sequencing data with CARD entries confirms that resistance genes identified correspond to known resistance mechanism in *H. pylori,* validating the molecular approach in this study [31,32].

The sequencing data provided additional insights into the genetic basis of resistance. Multiple mutations were identified in the *rdxA* gene (including R90K, A118T, C49T, T31E, Y62D, and V7I) all of which are known to reduce or abolish nitroreductase activity, thereby compromising metronidazole activation [26]. These mutations are clinically significant, as they result in high-level resistance and explain the treatment failure observed even in strains with seemingly functional *rdxA* alleles [26]. Similarly, the *frxA* gene mutations observed further support a loss-of-function model that contributes synergistically to metronidazole resistance [26,33,34].

For tetracycline, detection of mutations in the *16S rRNA* gene is consistent with known resistance mechanisms that block tetracycline’s access to the ribosomal binding site [24,25]. This is particularly important considering prior studies that showed low phenotypic resistance to tetracycline in Portugal [12,13,14], suggesting a possible increase in genotypically resistant strains or limitations of culture-based methods in previous work.

Regarding amoxicillin resistance, identification of mutations in the *Pbp1A* gene indicates alterations in penicillin-binding protein 1A, reducing *β*-lactam binding affinity [24,25].

For clarithromycin, mutations in the *23S rRNA* gene are well-established causes of macrolide resistance, as they interfere with antibiotic binding to the peptidyl transferase center [24,25]. Its presence is particularly impactful because clarithromycin is often a first-line therapy component [15,20]. Importantly, even low-frequency resistance mutations can lead to treatment failure, justifying the routine use of molecular diagnostics in pre-treatment decision-making [2,15,16].

The present study revealed notably high prevalence rates of AMR genes (ARGs) in *H. pylori*-positive samples from Algarve, particularly to metronidazole (*rdxA*: 83.3%, *frxA*: 66.7%), tetracycline (*16S rRNA* mutation: 66.7%), amoxicillin (*Pbp1A*: 27.8%) and clarithromycin (*23S rRNA*: 16.7%), drugs used as fist-line treatment [15,20]. These findings align with worldwide and regional concerns regarding *H. pylori* resistance trends, but our results also exceed many previously reported rates, especially for tetracycline and metronidazole.

This elevated detection of multiple ARGs also aligns with global meta-analyses by Savoldi et al. (2018) and Salahi-Niri et al. (2024), who reported regional variability but highlighted Southern Europe and developing countries as high-resistance zones [10,27].

Worldwide resistance rates reported by Salahi-Niri et al. (2024), across 28 countries, were 35.3% for metronidazole, 32.6% for clarithromycin, 2.1% for tetracycline, and 4.8% for amoxicillin [27]. Similarly, Gebreslassie et al. (2020) reported 39.7% for metronidazole, 27.2% for clarithromycin, 22.5% for levofloxacin, and 4.6% for amoxicillin [35]. Our results from Algarve region show substantially higher metronidazole and tetracycline resistance, and moderately higher amoxicillin resistance, but lower clarithromycin resistance compared to these studies [27,35].

When comparing our results with other Portuguese data from Lopo et al. (2018) and Viegas et al. (2024), which noted 25–42% resistance for metronidazole and clarithromycin and low resistance for amoxicillin and tetracyclines, the present study shows notably higher resistance to metronidazole and tetracyclines, consistent with a possible regional variation or emerging tendency [12,13]. Compared to the study by Almeida et al. (2014), which involved patients from another Portuguese region (the center of Portugal), clarithromycin resistance in Algarve is lower (21.4% vs. 17.7%, respectively) [14]. Our study also indicates that metronidazole resistance genes are markedly higher than the ones reported by Almeida et al. (2014), suggesting potential regional differences and/or an increasing trend over time [14]. Regarding tetracycline resistance, back in 2014, in the study by Almeida et al. (2014), it was rare (<1%), whereas our study reports a detection of the *16S rRNA* mutation in 66.7%, suggesting either a substantial increase in resistance or that previous molecular resistance was underreported [14]. The detection of *16S rRNA* mutation in two-thirds of the samples, despite previous low resistance rates to tetracyclines in Portugal [14], may also indicate the spread of resistance strains or horizontal gene transfer events, as seen in animal reservoirs [30]. Amoxicillin resistance also appears elevated in Algarve (27.8%) compared to the <1% of the reported values by Almeida et al. (2014), suggesting emerging resistance [14].

Regarding the gender disparity observed (100% of females showing resistance genes; significant association with *rdxA* gene *p* = 0.043) aligns with findings from Zhang et al. (2021), which documented higher resistance rates in females, particularly for metronidazole and clarithromycin in age-stratified groups [36]. These results are supported by other studies that also suggest that women may be more affected by AMR due to higher rates of antimicrobial prescriptions and an increased vulnerability to certain infections [37,38,39,40]. These may support evidence of biological, behavioral, or prescribing-pattern differences underlying gender-based disparities in AMR [24]. Although the association between *rdxA* and female sex was statistically significant (*p* = 0.043), this did not meet the Bonferroni-adjusted threshold (*p* < 0.01), and the corrected *p*-value was 0.215. Therefore, this finding should be interpreted with caution and considered exploratory.

The sample had a modest size and a gender imbalance (11 females vs. 7 males), which may limit the robustness of this result. Although statistically significant, the association may not be biologically meaningful without validation in larger, more balanced cohorts. Therefore, this result should be considered exploratory and hypothesis-generating rather than definitive. Future studies with larger sample sizes are needed to determine whether gender plays a consistent role in resistance gene prevalence.

The association of antibiotic resistance with consumption patterns, highlights how regional usage correlates with resistance trends. While Algarve-specific consumption data were not included, the high metronidazole resistance may reflect similar overuse or local prescribing habits that stress the need for further investigation [9].

The detection of multiple resistance genes in the majority of the samples (72.2%) raises important questions regarding prior antibiotic exposure and potential cumulative selective pressure, particularly for commonly used antibiotics such as metronidazole and tetracycline. While the current study did not have access to individual treatment histories or age stratification data, prior research suggests that multiple resistances often correlate with previous eradication failures or empirical antibiotic use [9,10]. Additionally, emerging evidence indicates that age and gender may influence resistance patterns, with older patients and females potentially being more prone to resistant strains due to higher lifetime antibiotic exposure [28,36]. Future prospective studies incorporating clinical metadata are warranted to better assess these associations.

These results underscore the importance of integrating local molecular surveillance into clinical practice, as real-time knowledge of resistance profiles can aid avoiding ineffective empirical treatments and reduce further resistance development, as endorsed by recent guidelines [2,15,16]. Given the resistance patterns observed in our study, levofloxacin-based regimens or rifabutin-containing rescue therapies could be considered, especially for patients with previous treatment failure [15]. Additionally, adjunctive use of probiotics, though not curative, has shown promise in improving eradication rates and reducing side effects, possibly by modulating the gastric microbiota and enhancing mucosal immunity [20,41]. The application of molecular testing to guide resistance-based therapy, as demonstrated in this study, is crucial for optimizing treatment outcomes in resistant *H. pylori* cases.

Despite the valuable preliminary data for the Algarve region, the sample size may be sufficient to identify trends or generate hypotheses but cannot provide high confidence or generalizability. The small sample size limits the statistical power and may affect the generalizability of the findings. It was conducted in a single geographical region which may not reflect AMR patterns in other parts of Portugal or Europe. These limitations highlight the need for future studies involving larger, multicentric approaches.

## 4. Materials and Methods

### 4.1. Sample Collection

DNA extracts from gastric samples of patients who underwent upper gastric endoscopy with gastric biopsy in Algarve, Portugal, containing *H. pylori* DNA shown by PCR and sequencing analysis, were analyzed for the presence of *H. pylori*-specific ARGs.

The gastric samples were collected at the endoscopy units of three hospitals in Algarve, Portugal (Unidade Local de Saúde do Algarve–Hospital de Faro and Hospital de Portimão and the Hospital Particular do Algarve–Unidade de Alvor), between December 2024 and February 2025. As described in a previous study by Cortez Nunes et al. 2025 [7].

To be included in the study, all patients had to be 18 years or older, sign a consent form and had to be off proton pump inhibitors and antimicrobials for at least 14 days and 30 days before the procedure, respectively. Informed written consent was obtained from all the participants.

### 4.2. Molecular Analysis

The *H. pylori* PCR-positive samples were subjected to conventional PCR assays to test for the presence of genes related to AMR in *H. pylori*, including *Pbp1A* (amoxicillin), *rdxA* and *frxA* (metronidazole), *16S rRNA* mutation gene (tetracycline) and *23S rRNA* (clarithromycin) to identify point mutations as described by Diab et al. 2018, Lee et al. 2018 and Cortez Nunes et al. 2023 using the primers and conditions describe in Table 3 [26,30,42].

The amplicons of each PCR-positive sample underwent bidirectional sequencing using the Sanger method at the Genomics Core facility of the Institute of Molecular Pathology and Immunology of the University of Porto, Portugal. Sequence editing and multiple alignments were performed with the MegaX Molecular Evolutionary Genetic Analysis version 10.1.8 [43]. The sequences obtained were subject to BLAST analysis using the non-redundant nucleotide database (http://blast.ncbi.nlm.nih.gov/Blast.cgi (accessed on 24 April 2025)) [44,45]. Sequences were also analyzed using tBLASTx through the Comprehensive Antibiotic Resistance Database (CARD) and the Resistance Gene Identifier (RGI) tool, applying the perfect, strict and loose hits to identify additional antibiotic-resistant gene mutations [31]. PCR products were considered positive only if the resulting amplicons showed significant sequence homology to known antimicrobial resistance genes upon alignment.

### 4.3. Statistical Analysis

The data were analyzed using IBM SPSS^®^ Statistics v30 (IBM Corp., Armonk, NY, USA). Frequencies and descriptive statistics were calculated and used to summarize gene prevalence and resistant patterns. For bivariate analysis, categorical variables (AMR genes and sex) Fisher’s Exact Test was applied (considering the expected cell count < 5) to verify the statistical significance between groups of data. *p*-values < 0.05 were considered to be statistically significant.

All variables in this study were categorical, including binary outcomes such as presence or absence of ARGs, sex (male/female) and types of resistance patterns (mono-, dual-, triple-, quadruple-resistance). Fisher’s Exact test was selected due to the small sample size and the presence of expected frequencies below 5 in contingency tables. This test provided an exact *p*-value for determining associations between categorical variables, such as between patient sex and specific ARGs. To control for the risk of type I error to multiple hypothesis testing, a Bonferroni correction was applied. For the five independent comparisons between sex and the presence of ARGs, the adjusted significance threshold was set at *p* < 0.01.

## 5. Conclusions

It can be concluded that there is a high prevalence of ARGs associated with *H. pylori* infections in the studied patients in Algarve, Portugal, despite the small study population. To the best of the authors’ knowledge, this is the first study addressing *H. pylori* AMR rates in Algarve.

Our findings revealed that 88.9% of the *h. pylori*-positive samples carried at least one ARG, with particularly high frequencies of resistance to metronidazole (83.3%) and tetracycline (66.7%). These results align with increasing global AMR trends and suggest that treatment strategies relying on these antibiotics may be less effective in Algarve.

By achieving the objective of detecting specific ARGs (*rdxA*, *frxA*, *16S rRNA*, *Pbp1A*, and *23S rRNA*) through PCR and sequencing, this study underscores the utility of molecular methods in guiding effective therapy. The identification of clinically relevant resistance mutations, such as those linked to clarithromycin and metronidazole resistance, further highlights the need for personalized treatment protocols.

Despite the exploratory nature of this study, it provides valuable insight into local resistance dynamics, highlighting metronidazole and tetracycline as the most impacted antimicrobials. Given that ineffective treatment can result in persistent infection, increased antibiotic exposure, and elevated gastric cancer risk, these findings have direct impact implications for human health and public heath policy. The findings, also call for the integration of molecular diagnostic tolls in routine clinical workflows and suggest that gender-specific trends in resistance may need to be considered in empirical treatment decisions.

Ideally, this study should be continued and/or replicated with a larger number of participants and in other regions of Portugal to obtain a better view of the overall Portuguese AMR rates associated with *H. pylori*, as well as determine if there are regional differences regarding rates of *H. pylori* with ARGs.

## Figures and Tables

**Table 1:** PCR results of the ARGs analysis by sex.

	Total	Male	Female	
***N***	18	7	11	
	*n (%)*	*n (%)*	*n (%)*	
**ARG positive**	16 (88.9%)	5 (71.4%)	11 (100%)	
**Amoxicillin** ***Pbp1A gene***	5 (27.8%)	1 (14.3%)	4 (36.4%)	
**Clarithromycin** ***23s rRNA gene***	3 (16.7%)	2 (28.6%)	1 (9.1%)	
**Metronidazole** ***rdxA gene***	15 (83.3%)	4 (57.1%)	11 (100%)	*p* = 0.043
**Metronidazole** ***frxA gene***	12 (66.7%)	4 (57.1%)	8 (72.7%)	
**Tetracycline** ***16s rRNA mutation gene***	12 (66.7%)	4 (57.1%)	8 (72.7%)	

**Table 2:** Resistance patterns among the 18 *H. pylori* DNA-positive samples.

Resistance Patterns	*n* (%)
**No resistance**	2 (11.1%)
**Mono resistance**	
Metronidazole	2 (11.1%)
Tetracycline	1 (5.6%)
**Dual resistance**	
Amoxicillin + Metronidazole	2 (11.1%)
Metronidazole + Tetracycline	6 (33.3%)
**Triple resistance**	
Amoxicillin + Metronidazole + Tetracycline	2 (11.1%)
Metronidazole + Tetracycline + Clarithromycin	2 (11.1%)
**Quadruple resistance**	
Amoxicillin + Metronidazole + Tetracycline + Clarithromycin	1 (5.6%)

**Table 3:** Primer sequences and thermocycling conditions for the detection of genes and mutation genes conferring resistance to antimicrobials.

Antimicrobials		Sequence	Target Gene	Thermo Cycle Conditions			Reference
				Temp. (°C)	Time	Nr. Cycles	
Amoxicillin	Forward	GCG ACA ATA AGA GTG GCA	*Pbp1A*	95 95 56 72 72	3′ 1′ 1′ 5′ 10′	35	[26,30,42]
	Reverse	TGC GAA CAC CCT TTT AAA T					
Metronidazole	Forward	AAT TTG AGC ATG GGG CAG A	*rdxA*	95 94 60 72 72	5′ 30″ 30″ 1′ 10′	35	[26,30,42]
	Reverse	GAA ACG CTT GAA AAC ACC CCT					
	Forward	TGG ATA TGG CAG CCG TTT A	*frxA*	95 95 58 72 72	5′ 30″ 30″ 1′ 10′	35	[26,30,42]
	Reverse	GGT TAT CAA AAA GCT AAC AGC G					
Tetracycline	Forward	CGG TCG CAA GAT TAA AAC	*16S rRNA* *mutation*	95 95 55 72 72	10′ 5″ 2″ 30″ 10′	45	[30,42]
	Reverse	GCG GAT TCT CTC AAT GTC					
Clarithromycin	Forward	TCA GTG AAA TTG TAG TGG AGG TGA AAA	*23S rRNA*	95 92 60 72 72	10′ 15″ 1′ 1′ 10′	40	[30,42]
	Reverse	CAG TGC TAA GTT GTA GTA AAG GTC CA					

## Data Availability

The data presented in this study are available on request from the corresponding author due to privacy and ethical reasons.

## Supplementary Materials

The following supporting information can be downloaded at https://www.mdpi.com/article/10.3390/antibiotics14080780/s1, Figure S1—Example of agarose gel electrophoresis of PCR products of *H. pylori* *rdxA* gene fragments. +, PCR products were considered positive only if the resulting amplicons showed significant sequence homology to known antimicrobial resistance genes upon alignment; CTR, DNA extracted from pure culture of 26695 strain was used as control; B, negative control consisted solely of mix solution; Figure S2—Example of agarose gel electrophoresis of PCR products of *H. pylori frxA* gene fragments. +, PCR products were considered positive only if the resulting amplicons showed significant sequence homology to known antimicrobial resistance genes upon alignment; CTR, DNA extracted from pure culture of 26695 strain was used as control; B, negative control consisted solely of mix solution; Figure S3—Example of agarose gel electrophoresis of PCR products of *H. pylori 16S rRNA* gene fragments. +, PCR products were considered positive only if the resulting amplicons showed significant sequence homology to known antimicrobial resistance genes upon alignment; CTR, DNA extracted from pure culture of 26695 strain was used as control; B, negative control consisted solely of mix solution; Figure S4—Example of agarose gel electrophoresis of PCR products of *H. pylori Pbp1A* gene fragments. +, PCR products were considered positive only if the resulting amplicons showed significant sequence homology to known antimicrobial resistance genes upon alignment; CTR, DNA extracted from pure culture of 26695 strain was used as control; B, negative control consisted solely of mix solution; Figure S5—Example of agarose gel electrophoresis of PCR products of *H. pylori 23s rRNA* gene fragments. +, PCR products were considered positive only if the resulting amplicons showed significant sequence homology to known antimicrobial resistance genes upon alignment; CTR, DNA extracted from pure culture of 26695 strain was used as control; B, negative control consisted solely of mix solution; Figure S6—Results of the sequencing alignment using CARD and the RGI. The figure displays the percentage identity, mutation positions, and resistance gene matches for the most common mutations detected regarding the *rdxA* gene. Figure S7—Results of the sequencing alignment using CARD and the RGI. The figure displays the percentage identity, mutation positions, and resistance gene matches for the most common mutations detected regarding the *rdxA* gene; Figure S8—Results of the sequencing alignment using CARD and RGI. The figure displays the percentage identity, mutation positions, and resistance gene matches for the most common mutations detected regarding the *frxA* gene; Figure S9—Results of the sequencing alignment using CARD and the RGI. The figure displays the percentage identity, mutation positions, and resistance gene matches for the most common mutations detected regarding the *frxA* gene; Figure S10—CARD alignment results for *Helicobacter pylori 16S rRNA* mutation gene conferring resistance to tetracycline; Figure S11—CARD alignment results for *Helicobacter pylori Pbp1A* gene mutations conferring resistance to amoxicillin.
