# Supplementary material for: Molecular Detection of Multiple Antimicrobial Resistance Genes in Helicobacter pylori-Positive Gastric Samples from Patients Undergoing Upper Gastrointestinal Endoscopy with Gastric Biopsy in Algarve, Portugal
Source: Antibiotics (Basel). 2025 Aug 1;14(8):780. doi: 10.3390/antibiotics14080780 (PMC12382745; doi:10.3390/antibiotics14080780)
Supplement: Supplementary file 1 [file antibiotics-14-00780-s001.zip › antibiotics-3785323-supplementary.pdf]

Supplementary material

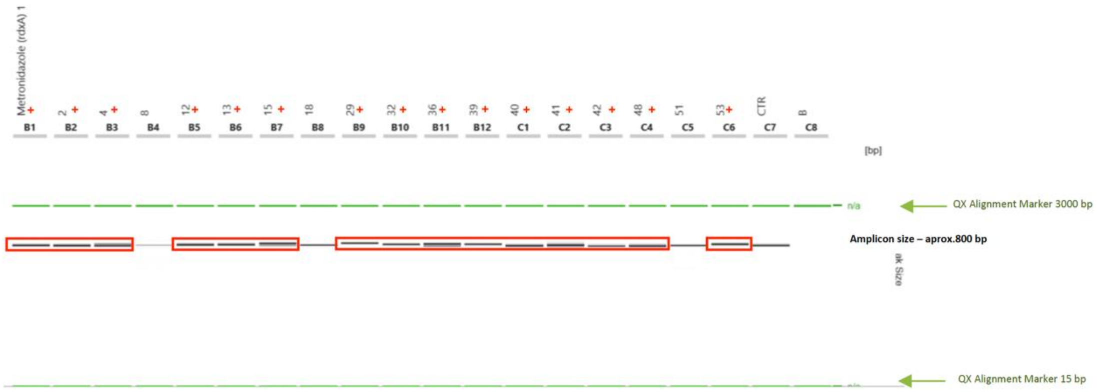

Figure S1. - Example of agarose gel electrophoresis of PCR products of *H. pylori* r*dxA* gene fragments. **+**, PCR products were considered positive only if the resulting amplicons showed significant sequence homology to known antimicrobial resistance genes upon alignment; **CTR**, DNA extracted from pure culture of 26695 strain was used as control; **B**, negative control consisted solely of mix solution.

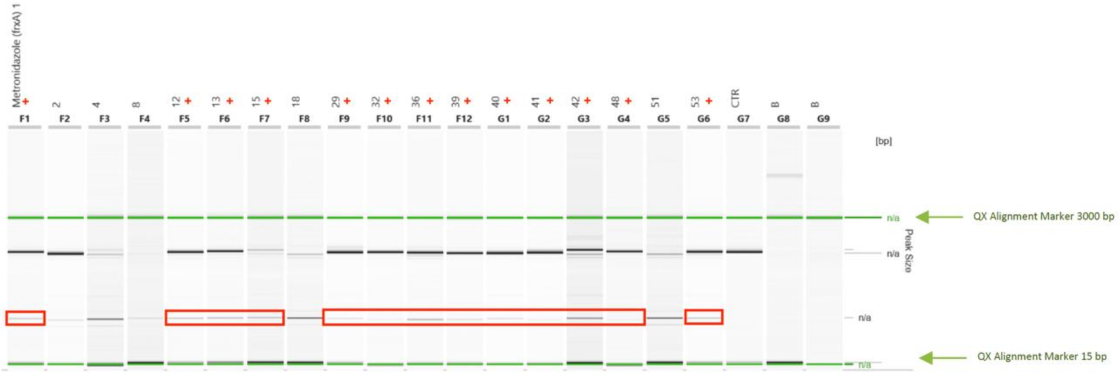

Figure S2. - Example of agarose gel electrophoresis of PCR products of *H. pylori frxA* gene fragments. **+**, PCR products were considered positive only if the resulting amplicons showed significant sequence homology to known antimicrobial resistance genes upon alignment; **CTR**, DNA extracted from pure culture of 26695 strain was used as control; **B**, negative control consisted solely of mix solution.

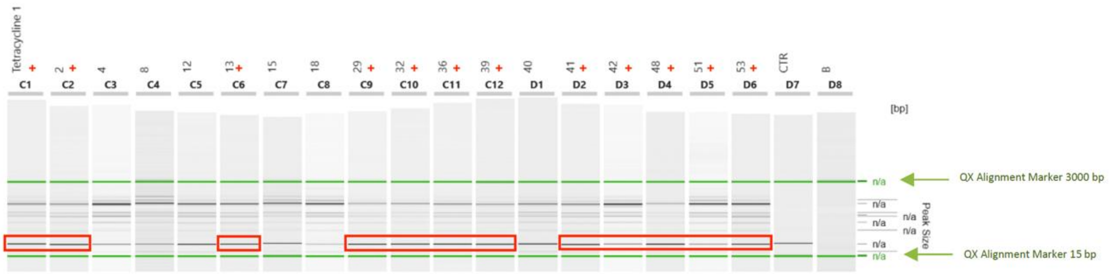

Figure S3. - Example of agarose gel electrophoresis of PCR products of *H. pylori 16S rRNA* gene fragments. **+**, PCR products were considered positive only if the resulting amplicons showed significant sequence homology to known antimicrobial resistance genes upon alignment; **CTR**, DNA extracted from pure culture of 26695 strain was used as control; **B**, negative control consisted solely of mix solution.

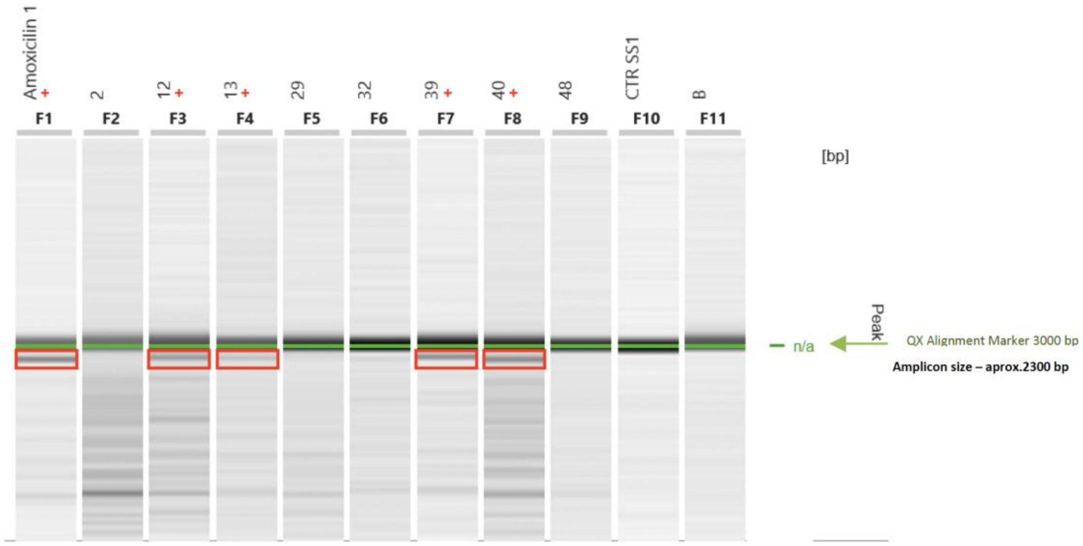

Figure S4. - Example of agarose gel electrophoresis of PCR products of *H. pylori Pbp1A* gene fragments. **+**, PCR products were considered positive only if the resulting amplicons showed significant sequence homology to known antimicrobial resistance genes upon alignment; **CTR**, DNA extracted from pure culture of 26695 strain was used as control; **B**, negative control consisted solely of mix solution.

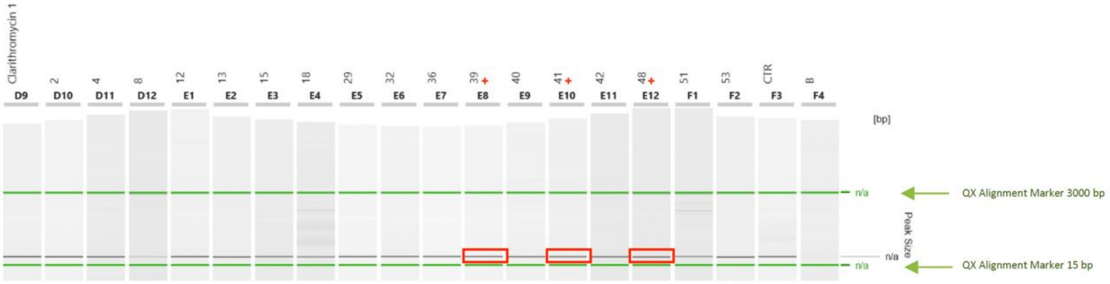

Figure S5. - Example of agarose gel electrophoresis of PCR products of *H. pylori 23s rRNA* gene fragments. **+**, PCR products were considered positive only if the resulting amplicons showed significant sequence homology to known antimicrobial resistance genes upon alignment; **CTR**, DNA extracted from pure culture of 26695 strain was used as control; **B**, negative control consisted solely of mix solution.

RGI Criteria	ARO Term	SNP	Detection Criteria	AMR Gene Family	Drug Class	Resistance Mechanism	% Identity of Matching Region	% Length of Reference Sequence	AST Source
Loose	Helicobacter pylori rdxA mutation conferring resistance to metronidazole	C49T, D59N	protein variant model	Antibiotic resistant Helicobacter pylori nitroreductase	nitroimidazole antibiotic	antibiotic target alteration	97.18	35.71	Curated-R
Loose	Helicobacter pylori rdxA mutation conferring resistance to metronidazole	H97T, P106S	protein variant model	Antibiotic resistant Helicobacter pylori nitroreductase	nitroimidazole antibiotic	antibiotic target alteration	93.33	64.29	Curated-R

Figure S6. - Results of the sequencing alignment using CARD and the RGI. The figure displays the percentage identity, mutation positions, and resistance gene matches for the most common mutations detected regarding the *rdxA* gene.

RGI Criteria	ARO Term	SNP	Detection Criteria	AMR Gene Family	Drug Class	Resistance Mechanism	% Identity of Matching Region	% Length of Reference Sequence	AST Source
Strict	Helicobacter pylori rdxA mutation conferring resistance to metronidazole	H97T, P106S, C49T	protein variant model	Antibiotic resistant Helicobacter pylori nitroreductase	nitroimidazole antibiotic	antibiotic target alteration	95.71	100.00	Curated-R

Figure S7. - Results of the sequencing alignment using CARD and the RGI. The figure displays the percentage identity, mutation positions, and resistance gene matches for the most common mutations detected regarding the *rdxA* gene.

RGI Criteria	ARO Term	SNP	Detection Criteria	AMR Gene Family	Drug Class	Resistance Mechanism	% Identity of Matching Region	% Length of Reference Sequence	AST Source
Strict	Helicobacter pylori frxA mutation conferring resistance to metronidazole	Y62D	protein variant model	Antibiotic resistant Helicobacter pylori nitroreductase	nitroimidazole antibiotic	antibiotic target alteration	100.0	99.08	Curated-R

Figure S8. - Results of the sequencing alignment using CARD and RGI. The figure displays the percentage identity, mutation positions, and resistance gene matches for the most common mutations detected regarding the *frxA* gene.

RGI Criteria	ARO Term	SNP	Detection Criteria	AMR Gene Family	Drug Class	Resistance Mechanism	% Identity of Matching Region	% Length of Reference Sequence	AST Source
Strict	Helicobacter pylori frxA mutation conferring resistance to metronidazole	V7I, Y62D	protein variant model	Antibiotic resistant Helicobacter pylori nitroreductase	nitroimidazole antibiotic	antibiotic target alteration	98.54	98.16	Curated-R

Figure S9. - Results of the sequencing alignment using CARD and the RGI. The figure displays the percentage identity, mutation positions, and resistance gene matches for the most common mutations detected regarding the *frxA* gene.

Bitscore	ARO tag	Name	Evalue	Identity	Species	Alignment
31	ARO:3003510	Helicobacter pylori 16S rRNA mutation conferring resistance to tetracycline	0.123041	100	Helicobacter pylori 26695	View

Figure S10. - CARD alignment results for *Helicobacter pylori 16S rRNA* mutation gene conferring resistance to tetracycline.

Bitscore	ARO tag	Name	Evalue	Identity	Species	Alignment
568	ARO:3007060	Helicobacter pylori pbp1 mutants conferring resistance to amoxicillin	0	98	Helicobacter pylori	View
530	ARO:3007060	Helicobacter pylori pbp1 mutants conferring resistance to amoxicillin	9.15044e-170	97	Helicobacter pylori	View
129	ARO:3007060	Helicobacter pylori pbp1 mutants conferring resistance to amoxicillin	6.31171e-132	94	Helicobacter pylori	View
191	ARO:3007060	Helicobacter pylori pbp1 mutants conferring resistance to amoxicillin	2.99434e-114	94	Helicobacter pylori	View

Figure S11. - CARD alignment results for *Helicobacter pylori Pbp1A* gene mutations conferring resistance to amoxicillin.
